# Hepatic and intestinal microcirculation and pulmonary inflammation in a model of veno-arterial extracorporeal membrane oxygenation in the rat

**DOI:** 10.1186/s40635-026-00938-w

**Published:** 2026-07-07

**Authors:** Fabian Edinger, Thomas Zajonz, Nico Mayer, Götz Schmidt, Emmanuel Schneck, Michael Sander, Christian Koch

**Affiliations:** Department of Anaesthesiology, Critical Care Medicine and Pain Therapy, University Hospital of Giessen, Justus-Liebig-University, Rudolf-Buchheim-Str.7, 35392 Giessen, Germany

**Keywords:** V-A ECMO, Inflammation, Intestinal microcirculation, Hepatic microcirculation

## Abstract

**Background:**

Due to peripheral cannulation, veno-arterial (V-A) extracorporeal membrane oxygenation (ECMO) therapy is increasingly used outside the cardiac operation room. Despite significant efforts toward improving therapy for septic shock, mortality remains high. In this context, V-A ECMO therapy remains controversial and has been associated with reduced intestinal microcirculation in a septic rat model. In addition to ECMO-induced inflammation, peripheral V-A ECMO therapy is associated with lower-body hyperoxia. Therefore, this study investigated the impact of V-A ECMO therapy on intestinal microcirculation and systemic and pulmonary inflammation in healthy rats without septic shock.

**Methods:**

Thirty male Lewis rats were randomly assigned to three groups: sham, low-flow (60 mL/kg/min) and high-flow (90 mL/kg/min). V-A ECMO was established by jugular venous drainage and femoral arterial return. Intestinal and hepatic microcirculation were assessed by micro-light guide spectrophotometry after median laparotomy. Systemic and pulmonary inflammation were evaluated by measuring plasma and bronchoalveolar lavage (BAL) levels of tumour necrosis factor (TNF-α), interleukins 6 (IL-6) and 10 (IL-10), and C-X-C motif chemokine ligands 2 (CXCL-2) and 5 (CXCL-5). Hemodynamic data were captured using a left ventricular pressure–volume catheter.

**Results:**

Intestinal oxygenation did not differ significantly among groups (sham: 76%, low-flow: 77%, high-flow: 74%; all *p* > 0.05). In contrast, hepatic oxygenation was significantly lower during V-A ECMO therapy (sham: 43%, low-flow: 23%, high-flow: 27%; all *p* ≤ 0.001). TNF-α levels were significantly elevated during both low-flow (*p* = 0.027) and high-flow (*p* = 0.015) V-A ECMO therapy. In contrast, IL-10 levels were significantly reduced during low-flow (*p* = 0.009) but not high-flow (*p* = 1.000) V-A ECMO therapy, whereas IL-6 levels were not significantly affected (low-flow: *p* = 0.764, high-flow: *p* = 0.104). BAL analysis revealed significantly reduced IL-6 levels during high-flow (*p* = 0.010) but not low-flow (*p* = 0.288) V-A ECMO therapy, whereas CXCL-2 (low-flow: *p* = 1.000, high-flow: *p* = 0.634) and CXCL-5 (low-flow: *p* = 1.000, high-flow: *p* = 0.224) levels were not significantly affected.

**Conclusions:**

In healthy rats, V-A ECMO therapy did not impair intestinal microcirculation and was associated with reduced pulmonary inflammation at the high flow rate, likely due to decreased pulmonary blood flow.

**Supplementary Information:**

The online version contains supplementary material available at 10.1186/s40635-026-00938-w.

## Background

Veno-venous (V-V) extracorporeal membrane oxygenation (ECMO) therapy has gained wide acceptance for treating critically ill patients over the last two decades due to the experiences and evidence gained during the two large respiratory virus pandemics in 2009 (influenza) and 2020 (severe acute respiratory syndrome coronavirus 2) [1–4]. In contrast, veno-arterial (V-A) ECMO therapy has been restricted to cardiac surgery for many years. Interestingly, V-A ECMO therapy has been increasingly used outside the cardiac operation room over the last decade, driven by the increasing use of femoral cannulation for V-A ECMO return. Peripheral V-A ECMO therapy is being applied during cardiogenic shock, pulmonary embolism, or circulatory arrest for extracorporeal cardiopulmonary resuscitation.

It is well established that the ECMO circuit and the large foreign surface of the ECMO membrane are associated with endothelial damage and activation of the inflammatory, complement, and coagulation systems [5, 6]. Since most human studies were performed during cardiopulmonary bypass (CPB), there is a knowledge gap between patients on CPB and ECMO [7]. It has to be noted that there are fundamental differences between CPB and V-A ECMO therapy: CPB is primarily employed during surgical procedures, with the majority of patients undergoing elective interventions. In contrast, V-A ECMO therapy is initiated in the intensive care setting in critically ill patients, frequently presenting with cardiogenic shock. Consequently, these patients often exhibit pre-existing immune dysregulation prior to support initiation, and V-A ECMO therapy may therefore constitute an additional inflammatory stimulus (“second hit”), in contrast to CPB [7]. Furthermore, CPB is generally limited to short-term application (typically 2–3 h), whereas the median duration of V-A ECMO support is approximately 4 days [8]. Another relevant distinction relates to circuit design: conventional CPB systems commonly incorporate an open venous reservoir with direct blood–air interface and cardiotomy suction, both of which are largely absent in V-A ECMO circuits. These features have been associated with enhanced activation of neutrophils and increased cytokine release [9]. Therefore, additional studies are needed including patients during V-A ECMO therapy.

This ECMO-induced inflammation is challenging to investigate in humans due to the heterogeneity of critically ill patients. Therefore, applying V-A ECMO therapy in inbred rat models offers the opportunity to obtain reproducible results.

During V-A ECMO therapy, blood flow through the lungs is reduced due to drainage from the right atrium. In our previous study using a rat model of septic shock and low blood flow, V-A ECMO therapy resulted in increased pulmonary inflammation [10]. However, the distinction between ECMO-induced and sepsis-induced pulmonary inflammation remained unclear.

Additionally, the femoral return from the V-A ECMO system is associated with a retrograde flow of highly oxygenated blood, creating a mixing zone between the ECMO return and cardiac output (CO) [11, 12]. The location of this mixing zone depends on the CO and the flow rate of the ECMO system [12]. Particularly with high ECMO flow rates, the mixing zone is located in the aortic arch, leading to lower body hyperoxia [13]. It is well established that hyperoxia is associated with the production of reactive oxygen species, cell death and intestinal barrier disruption [13, 14]. To examine the impact of hyperoxia on the intestine, our previous study investigated the intestinal and hepatic microcirculation in rat models of septic shock during V-A ECMO therapy [10]. Interestingly, V-A ECMO therapy was associated with reduced intestinal and hepatic microcirculation compared to sham procedure, independent of the blood flow rate. However, the impact of ECMO-induced inflammation on intestinal and hepatic microcirculation during V-A ECMO therapy in the absence of septic shock remains unclear. Therefore, this study’s primary aim was to evaluate intestinal and hepatic microcirculation in healthy rats undergoing V-A ECMO therapy with femoral return. Its secondary aim was to investigate the inflammatory response in the lungs during V-A ECMO therapy.

## Results

### Intestinal and hepatic microcirculation

**Fig. 1:**
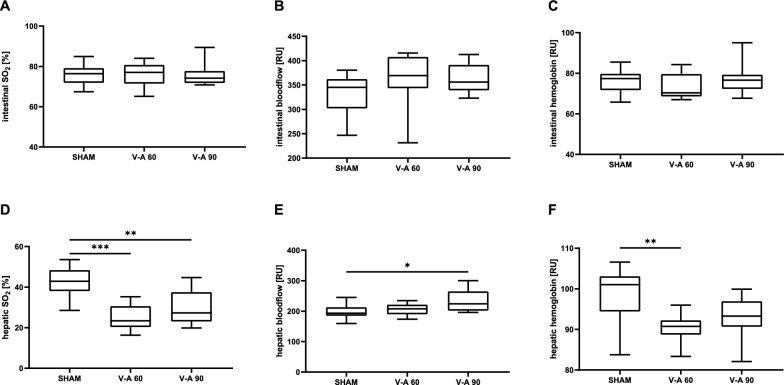
Means of the time-course of intestinal **(A)** regional oxygen saturation, **(B)** relative blood flow and **(C)** relative haemoglobin concentration, and hepatic **(D)** regional oxygen saturation, **(E)** relative blood flow, and **(F)** relative haemoglobin concentration. Intestinal regional tissue oxygen saturation (SO_2_), relative blood flow and relative haemoglobin concentration did not differ significantly between V-A 60, V-A 90, and sham procedure. Hepatic SO_2_ was significantly reduced during V-A 60 and V-A 90 ECMO therapy compared to sham group. While hepatic blood flow was faster only during V-A 90 ECMO therapy, only rats treated with V-A 60 ECMO therapy had a reduced hepatic relative haemoglobin concentration compared to sham animals (*n* = 10 per group). The asterisks denote the degree of statistical significance: *, *p* < 0.05; **, *p* < 0.01; ***, *p* < 0.001. Box and whisker plots indicate the median, interquartile range (box), and minimum and maximum (whiskers). Abbreviations: ECMO = extracorporeal membrane oxygenation; RU = relative units; SO_2_ = tissue oxygen saturation; V-A = veno-arterial

Intestinal tissue oxygen saturation (SO₂), relative blood flow and relative haemoglobin concentration assessed by white light and laser Doppler spectrometry were unchanged during low-flow (60 mL/kg/min, V-A 60) and high-flow (90 mL/kg/min, V-A 90) V-A ECMO therapy compared to sham group (all *p* > 0.05, Fig. 1A–C). Hepatic SO₂ was reduced during both V-A 60 and V-A 90 ECMO therapy compared to sham procedure (all *p* ≤ 0.001; Fig. 1D). In contrast, hepatic relative haemoglobin concentration was lower only during V-A 90 ECMO therapy (V-A 60: *p* = 0.003; V-A 90: *p* = 0.064; Fig. 1F). Similarly, hepatic relative blood flow was faster only during V-A 90 ECMO therapy (V-A 60: *p* = 1.000; V-A 90: *p* = 0.032; Fig. 1E). All medians and interquartile ranges are tabled in supplemental Table 1.

**Table 1:** Results of the blood gas analysis

		0 min	30 min	60 min	90 min	120 min
S_a_O_2_ [%]	sham	100 [100, 100]	100 [100, 100]	100 [100, 100]	100 [99, 100]	100 [99, 100]
	V-A 60	100 [100, 100]	100 [100, 100]	100 [100, 100]	100 [100, 100]	100 [100, 100]
	V-A 90	100 [100, 100]	100 [100, 100]	100 [100, 100]	100 [100, 100]	100 [100, 100]
S_cv_O_2_ [%]	sham	75 [68, 77]	78 [74, 82]	79 [75, 84]	77 [77, 80]	75 [73, 82]
	V-A 60	78 [72, 79]	85 [82, 89]	79 [74, 83]	76 [74, 81]	75 [71, 78]
	V-A 90	75 [73, 80]	80 [76, 88]	78 [71, 83]	74 [70, 81]	69 [63, 79]
pO_2_ [mmHg]	sham	141 [124, 156]	128 [127, 140]	130 [116, 132]	121 [107, 124]	114 [105, 125]
***	V-A 60	143 [128, 161]	233 [204, 247]	229 [216, 243]	219 [180, 241]	210 [200, 236]
***	V-A 90	135 [121, 165]	214 [196, 237]	211 [165, 216]	216 [194, 228]	196 [184, 213]
pCO_2_ [mmHg]	sham	42 [40, 44]	43 [42, 45]	42 [38, 44]	43 [40, 44]	42 [41, 44]
***	V-A 60	41 [38, 42]	37 [35, 40]	37 [35, 40]	37 [36, 39]	37 [36, 39]
***	V-A 90	42 [40, 44]	39 [36, 44]	38 [37, 40]	37 [35, 39]	38 [36, 39]
pH	sham	7.39 [7.36, 7.41]	7.39 [7.36, 7.42]	7.39 [7.35, 7.41]	7.36 [7.35, 7.41]	7.36 [7.35, 7.39]
***	V-A 60	7.38 [7.35, 7.42]	7.43 [7.41, 7.45]	7.44 [7.42, 7.46]	7.43 [7.41, 7.45]	7.44 [7.42, 7.45]
***	V-A 90	7.37 [7.33, 7.37]	7.41 [7.39, 7.45]	7.43 [7.41, 7.44]	7.41 [7.40, 7.44]	7.43 [7.41, 7.44]
BE	sham	0.4 [− 1.8, 0.9]	− 0.2 [− 1.2, − 1.6]	− 0.6 [− 1.8, 0.4]	− 1.2 [− 1.8, 0.7]	− 0.9 [− 1.3, − 0.3]
	V-A 60	− 0.7 [− 1.8, 0.6]	0.9 [− 0.4, 1.3]	1.6 [0.0, 2.0]	0.1 [− 0.6, 2.2]	1.5 [− 0.1, 2.2]
	V-A 90	− 1.9 [− 2.5, − 0.6]	0.6 [0.0, 1.5]	1.1 [− 0.6, 1.8]	− 0.2 [− 1.1, 1.1]	0.8 [− 0.9, 1.7]
Lac [mmol/L]	sham	1.3 [1.1, 1.8]	0.8 [0.6, 1.0]	0.8 [0.7, 0.9]	1.0 [0.9, 1.1]	0.9 [0.9, 1.1]
	V-A 60	1.6 [1.2, 1.7]	1.1 [1.0, 1.4]	0.9 [0.7, 1.1]	1.3 [0.9, 1.3]	1.2 [0.8, 1.2]
	V-A 90	1.2 [1.1, 1.5]	1.0 [0.9, 1.1]	0.9 [0.8, 1.0]	1.3 [1.1, 1.6]	1.3 [0.9, 1.4]
Hb [g/dL]	sham	13.8 [12.5, 14.3]	12.2 [11.4, 13.1]	12.0 [10.8, 12.6]	10.9 [9.6, 11.8]	10.8 [9.1, 11.5]
***	V-A 60	14.4 [13.8, 14.6]	8.0 [7.8, 8.2]	8.1 [7.9, 8.3]	7.6 [7.2, 7.9]	7.4 [7.2, 7.9]
***	V-A 90	13.3 [12.9, 14.3]	7.7 [7.2, 7.9]	7.5 [7.3, 7.9]	7.1 [7.0, 7.3]	7.1 [6.6, 7.7]
Hct [%]	sham	42.3 [38.4, 43.7]	37.5 [35.1, 40.4]	36.9 [33.2, 38.5]	33.7 [29.6, 36.5]	33.4 [28.4, 35.3]
***	V-A 60	44.1 [42.2, 44.9]	24.8 [23.9, 25.7]	25.2 [24.6, 25.7]	23.6 [22.5, 24.7]	23.0 [22.5, 24.2]
***	V-A 90	40.9 [39.5, 43.8]	23.9 [22.5, 24.6]	23.2 [22.6, 24.5]	22.3 [21.7, 22.8]	22.2 [20.6, 23.9]
Glu [mg/dL]	sham	166 [161, 178]	142 [132, 149]	129 [124, 134]	127 [124, 131]	126 [117, 137]
	V-A 60	172 [157, 184]	141 [132, 145]	124 [122, 132]	122 [115, 130]	117 [114, 130]
	V-A 90	164 [149, 172]	142 [132, 146]	132 [112, 136]	122 [113, 130]	121 [115, 129]
Na [mmol/L]	sham	141 [141, 143]	141 [141, 143]	142 [141, 143]	142 [142, 144]	143 [142, 144]
**	V-A 60	143 [141, 144]	143 [142, 144]	144 [143, 144]	144 [144, 145]	144 [144, 145]
	V-A 90	143 [142, 143]	142 [142, 143]	143 [142, 144]	143 [143, 143]	143 [143, 144]
K [mmol/L]	sham	4.0 [3.9, 4.1]	4.4 [4.3, 4.5]	4.2 [4.1, 4.4]	4.2 [4.2, 4.4]	4.3 [4.1, 4.4]
	V-A 60	4.2 [3.9, 4.4]	4.2 [4.0, 4.3]	4.2 [4.0, 4.3]	4.3 [4.2, 4.3]	4.4 [4.2, 4.6]
	V-A 90	4.1 [4.0, 4.2]	4.1 [4.0, 4.1]	4.3 [4.1, 4.3]	4.3 [4.1, 4.4]	4.4 [4.2, 4.5]
Cl [mmol/L]	sham	108 [107, 109]	109 [107, 111]	110 [108, 111]	111 [110, 111]	111 [110, 113]
**	V-A 60	109 [108, 110]	112 [110, 112]	112 [112, 113]	113 [112, 114]	114 [113, 114]
	V-A 90	109 [107, 110]	111 [109, 112]	112 [110, 113]	113 [111, 113]	113 [111, 114]
Ca [mmol/L]	sham	1.49 [1.47, 1.53]	1.49 [1.47, 1.53]	1.50 [1.49, 1.53]	1.49 [1.49, 1.53]	1.50 [1.48, 1.51]
	V-A 60	1.50 [1.49, 1.52]	1.49 [1.47, 1.50]	1.47 [1.46, 1.49]	1.48 [1.44, 1.50]	1.47 [1.42, 1.50]
	V-A 90	1.52 [1.50, 1.55]	1.48 [1.48, 1.49]	1.49 [1.48, 1.52]	1.49 [1.46, 1.51]	1.48 [1.46, 1.50]

Data are presented as the median [interquartile range]. The degree of statistical significance between sham and V-A ECMO 60 or V-A ECMO 90 is denoted by asterisks *, *p* < 0.05; **, *p* < 0.01; ***, *p* < 0.001. BE = base excess; Ca = calcium; Cl = chloride; Glu = glucose; Hb = haemoglobin; Hct = haematocrit; K = potassium; Lac = lactate; Na = sodium; pCO_2_ = arterial partial pressure of carbon dioxide; pO_2_ = arterial partial pressure of oxygen; S_a_O_2_ = arterial oxygen saturation; S_cv_O_2_ = central venous oxygen saturation; V-A = veno-arterial

### Hemodynamic measurements

**Fig. 2:**
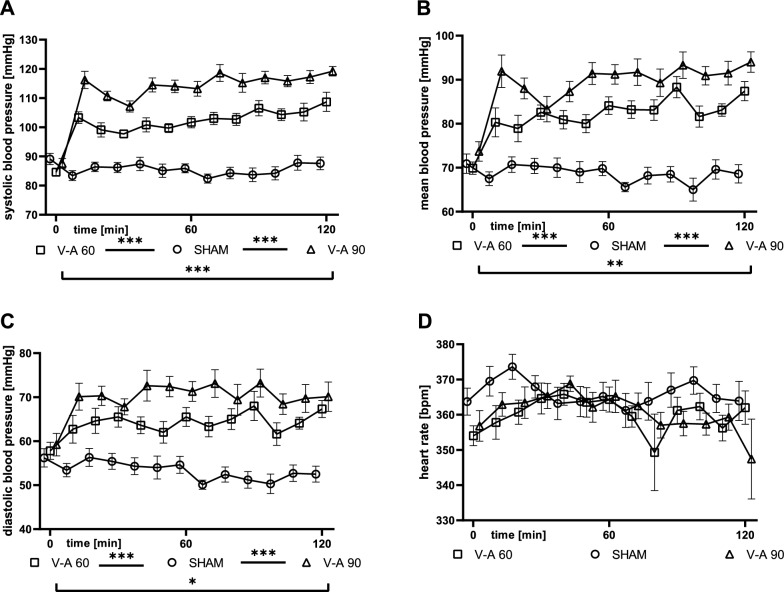
Time course of **(A)** SAP, **(B)** MAP, **(C)** DAP and **(D)** heart rate. SAP was higher during V-A 90 ECMO therapy compared to V-A 60 ECMO therapy and sham procedure. MAP, DAP and heart rate did not differ significantly between all groups (*n* = 10 per group). The asterisks denote the degree of statistical significance: *, *p* < 0.05; **, *p* < 0.01; ***, *p* < 0.001. Abbreviations: DAP = diastolic arterial pressure; ECMO = extracorporeal membrane oxygenation; MAP = mean arterial pressure; SAP = systolic arterial pressure; V-A = veno-arterial

Systolic (SAP), mean (MAP) and diastolic (DAP) arterial blood pressures were higher during both V-A 60 and 90 ECMO therapy compared to sham procedure (all *p* < 0.001, Fig. 2A–C). Additionally, SAP, MAP and DAP were higher during V-A 90 ECMO therapy than during V-A 60 ECMO therapy (SAP: *p* < 0.001; MAP: *p* = 0.001; DAP: *p* = 0.024). However, heart rate did not differ significantly between V-A 60, V-A 90 and sham group (Fig. 2D).

**Fig. 3:**
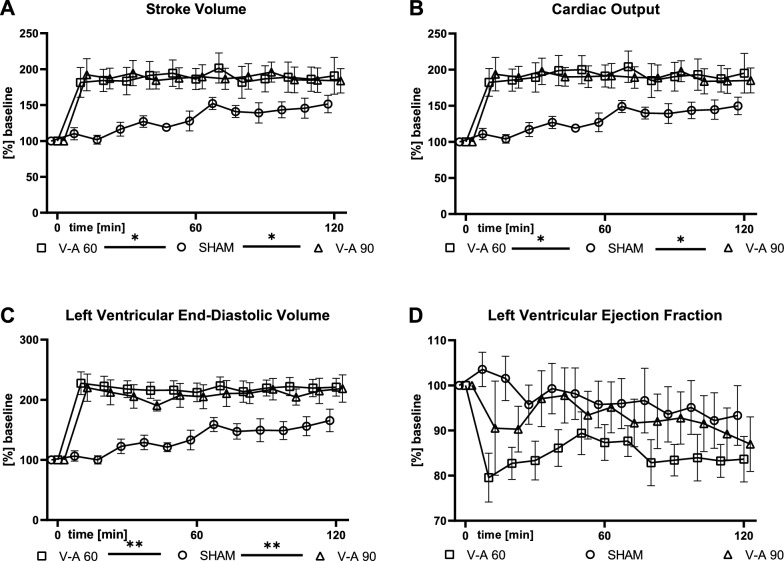
Time course of **(A)** SV, **(B)** CO, **(C)** LVEDV and **(D)** LVEF. SV, CO, and LVEDV were significantly higher during both V-A 60 and VA-90 ECMO therapy compared to sham procedure. LVEF did not differ significantly between the groups (*n* = 10 per group). The asterisks denote the degree of statistical significance: *, *p* < 0.05; **, *p* < 0.01. Abbreviations: CO = cardiac output; ECMO = extracorporeal membrane oxygenation; LVEDV = left ventricular end-diastolic volume; LVEF = left ventricular ejection fraction; SV = stroke volume; V-A = veno-arterial

Analysis of the conductance catheter data showed elevated stroke volume (SV), CO, and left ventricular end-diastolic volume (LVEDV) during V-A 60 and V-A 90 ECMO therapy compared to sham procedure (SV – V-A 60: *p* = 0.026, V-A 90: *p* = 0.025; CO – V-A 60: *p* = 0.019, V-A 90: *p* = 0.023; LVEDV – V-A 60: *p* = 0.001, V-A 90: *p* = 0.005; Fig. 3). However, left ventricular ejection fraction (LVEF, Fig. 3D) and left ventricular end-diastolic pressure (LVEDP) did not differ significantly between the groups (LVEF Fig. 3D, supplemental Fig. S1).

### Blood gas analysis

While arterial (S_a_O_2_) and central venous (S_cv_O_2_) oxygen saturation did not differ between the groups, arterial partial pressures of oxygen (pO_2_) were higher during both V-A 60 and V-A 90 ECMO therapy compared to sham procedure (all *p* < 0.001, Table 1). In contrast, arterial partial pressure of carbon dioxide (pCO_2_) was significantly lower during V-A 60 and V-A 90 ECMO therapy than during sham procedure, whereas pH was significantly higher (all *p* < 0.001, Table 1). Additionally, haemoglobin concentrations and haematocrit values were lower during both V-A 60 and V-A 90 ECMO therapy than during sham procedure (all *p* < 0.001). However, sodium and chloride concentrations were only elevated during V-A 60 ECMO therapy (*p* = 0.004 and 0.004, respectively; Table 1). Notably, lactate, base excess (BE), glucose, potassium and calcium concentrations did not differ significantly between the groups (Table 1).

### Inflammatory parameters

**Fig. 4:**
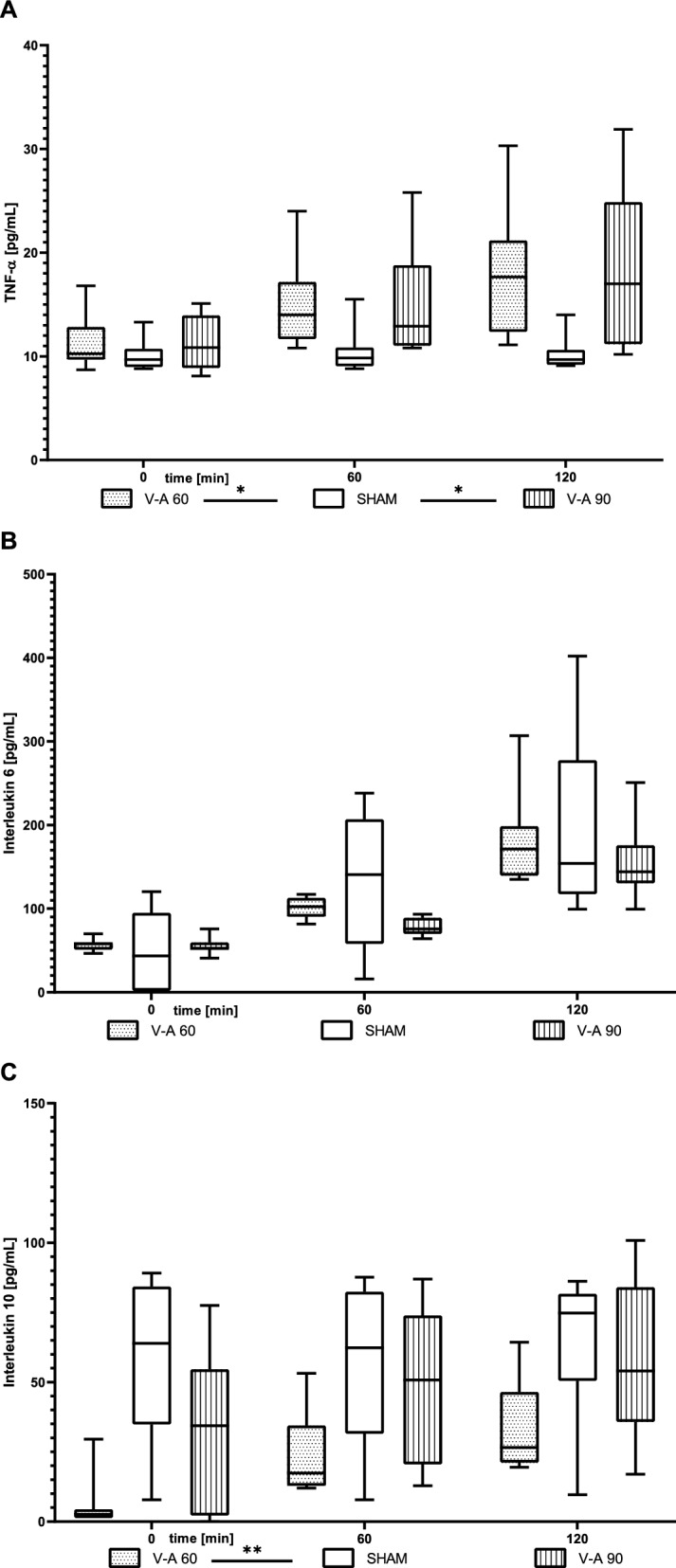
Time course of the plasma concentrations of the inflammatory parameters **(A)** TNF-α, **(B)** IL-6 and **(C)** IL-10. While TNF-α concentrations were significantly higher during both V-A 60 and V-A 90 ECMO therapy than during sham procedure, IL-10 levels were only lower during V-A 60 ECMO therapy. However, IL-6 levels did not differ significantly between the groups (*n* = 10 per group). The asterisks denote the degree of statistical significance: *, *p* < 0.05; **, *p* < 0.01. Box and whisker plots indicate the median, interquartile range (box), and minimum and maximum (whiskers). Abbreviations: TNF-α = tumor necrosis factor-alpha; IL-10 = interleukin 10; IL-6 = interleukin 6

Tumour necrosis factor (TNF-α) concentrations were significantly higher during both V-A 60 and V-A 90 ECMO therapy than during sham procedure (*p* = 0.027 and 0.015, respectively). In contrast, interleukin 6 (IL-6) concentrations did not differ significantly between the groups (Fig. 4). Additionally, interleukin 10 (IL-10) concentrations were significantly lower during V-A 60 ECMO therapy (*p* = 0.009) but not during V-A 90 ECMO therapy (*p* = 1.000) compared to sham group. Notably, the concentrations of C-X-C motif chemokine ligands 2 (CXCL-2) and 5 (CXCL-5) were below the detection limit of the enzyme-linked immunosorbent assay (ELISA).

**Fig. 5:**
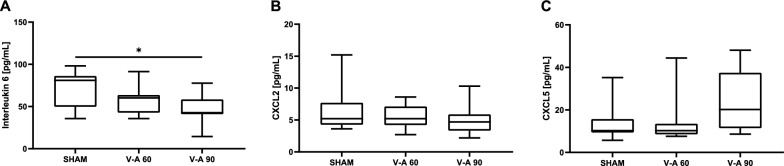
The concentrations of the pro-inflammatory cytokines **(A)** IL-6, **(B)** CXCL-2 and **(C)** CXCL-5 in BAL. IL-6 concentrations were significantly lower during V-A 90 ECMO therapy compared to sham procedure. However, CXCL-2 and CXCL-5 concentrations did not differ significantly between the groups (*n* = 10 per group). The asterisks denote the degree of statistical significance: *, *p* < 0.05. Box and whisker plots indicate the median, interquartile range (box), and minimum and maximum (whiskers). Abbreviations: BAL = broncho alveolar lavage; CXCL-2 = C-X-C motif chemokine ligand 2; CXCL-5 = C-X-C motif chemokine ligand 5; IL-6 = interleukin 6; V-A = veno-arterial

Analysis of the broncho alveolar lavage (BAL) revealed reduced IL-6 levels during V-A 90 ECMO therapy (*p* = 0.010) but not during V-A 60 ECMO therapy (*p* = 0.288) compared to sham group (Fig. 5). However, CXCL-2 and CXCL-5 levels in BAL did not differ significantly between the groups (Fig. 5). Notably, the levels of TNF-α and IL-10 in BAL were below the detection limit of the ELISA.

## Discussion

To the best of our knowledge, this study is the first to describe an in vivo model of intestinal and hepatic microcirculation during V-A ECMO therapy in healthy rats with jugular drainage and femoral return. In brief, our study revealed three main findings. Firstly, although no differences in intestinal microcirculation were observed, rats that received V-A ECMO therapy showed reduced hepatic microcirculation. Secondly, SAP, MAP, DAP, SV, CO and LVEDV increased during V-A ECMO therapy. Thirdly, plasma TNF-α levels were elevated, and plasma IL-10 levels were reduced during V-A ECMO therapy. Moreover, IL-6 levels in BAL were reduced during V-A ECMO therapy.

Since assessing the intestinal microcirculation is complicated in critically ill humans and abdominal complications are common during femoral V-A ECMO therapy [15], our findings are of great importance. Furthermore, the Extracorporeal Life Support Organisation (ELSO) has published a consensus statement on nutrition during ECMO therapy, which also supports the relevance of our study [16]. Previously, our group analysed intestinal and hepatic microcirculation during V-A ECMO therapy in septic rats [10]. Interestingly, we found reduced intestinal SO_2_ and relative haemoglobin concentration. However, it remained unclear whether these changes were caused by V-A ECMO therapy alone or by the combination of V-A ECMO therapy and septic shock. Therefore, we examined the impact of V-A ECMO in healthy rats in this study. We observed no differences between rats receiving sham and V-A ECMO therapy, indicating that V-A ECMO therapy does not affect the intestinal microcirculation in healthy rats. Thus, it appears that the combination of V-A ECMO therapy and septic shock impaired intestinal microcirculation in our previous study, consistent with a second-hit model [10]. Since these findings in septic animals were not validated histological or by biomarkers, this conclusion remains speculative.

Since no studies have examined the intestinal microcirculation during V-A ECMO therapy in human patients, our findings can be compared only with those from human patients undergoing cardiopulmonary bypass. For instance, Thorén et al. measured an increased jejunal microcirculation during mild hypothermic cardiopulmonary bypass using Laser Doppler flowmetry [17]. Nevertheless, as previously described, the use of a heart–lung machine with suction pumps, aortic cross-clamping and central cannulation is not comparable to peripheral cannulation in the V-A ECMO approach used in this study [7].

Critically ill patients often show dysbiosis with depletion of beneficial and overgrowth of harmful bacteria [18]. Dysbiosis has been associated with sepsis, multiorgan failure, and nosocomial infections [19]. In particular, hyperoxia is associated with dysbiosis [20]. Therefore, femoral V-A ECMO patients are at a high risk of abdominal complications.

Interestingly, the reduced hepatic microcirculation observed in healthy rats in this study is consistent with our previous results using septic animals [10]. Thus, these effects do not appear to be due to lipopolysaccharide administration to induce septic shock. Since the draining cannula is positioned in the right atrium, it should not have affected the hepatic microcirculation. Moreover, an impaired hepatic microcirculation has also been reported in healthy or septic rats undergoing femoral jugular V-V ECMO therapy [21, 22]. Notably, the hepatic probe is designed with an adhesive surface, which could have reduced local perfusion, potentially explaining the reduction in hepatic microcirculation. Following this, a reduced hepatic microcirculation would have been measured in the sham group as well. It should be noted that the blood of the rats was diluted with the priming volume of the ECMO circuit. During high flow V-A ECMO therapy the haemoglobin concentration declined from 13.3 to 7.7 g/dL. Therefore, the reduced hepatic SO_2_ might reflect the anaemia.

Next, the macrocirculation was analysed. V-A ECMO therapy was found to increase arterial blood pressure in healthy rats in this study, consistent with our previous studies using V-A ECMO therapy in healthy or septic rats [10, 23, 24]. Interestingly, this study demonstrated that the increase in arterial blood pressure was flow-dependent, as V-A 60 ECMO therapy was associated with significantly lower blood pressure than V-A 90 ECMO therapy. These results demonstrate that our rat model is working well.

Additionally, the increased SV, CO and LVEDV are also consistent with our previous studies [10, 23, 24]. The retrograde return of oxygenated blood increases afterload, and the conductance catheter is placed into the left ventricle through the aortic valve, resulting in aortic regurgitation. These facts may explain the increased LVEDV. Nevertheless, complete unloading of the heart should have resulted in a reduced CO measured in the left ventricle, with reduced antegrade blood flow. Contrary to this, a pulsatile flow curve in the tail artery was observed throughout the experiments. The higher blood flow of 90 mL/kg/min was derived from human extracorporeal support. However, since the internal jugular vein was stretched by the draining cannula, no lager cannula could be used in our setup, and the blood flow could not be increased further. Connelly et al. measured CO in healthy Sprague–Dawley rats and reported a CO of 67.8 mL/kg/min. Nevertheless, it can be assumed that the native CO in our study exceeded 90 mL/kg/min, which may explain the persistent antegrade blood flow. In addition, dilution anaemia was observed in all rats undergoing V-A ECMO therapy due to the large priming volume of the circuit. Notably, anaemia is associated with increased SV and CO, which may also explain the increased SV and CO observed in this study [25]. Since no increase in lactate levels was observed, the critical haemoglobin concentration seems not to have been reached. Although it failed to reach statistical significance, a reduction in LVEF together with an increase in LVEDP can be seen after commencing ECMO. The reduced LVEF could be explained by the increased LVEDP. In addition, the systolic arterial blood pressure rose acutely upon ECMO initiation. Although the circuit’s priming volume had been minimized, substantial haemodilution still occurred, reducing midazolam and fentanyl concentrations. While all animals received an anaesthetic bolus at ECMO onset to mitigate this effect, insufficient anaesthesia levels might still have persisted. This may offer a plausible explanation for the subsequent increase in LVEDP and decrease in LVEF. Despite lung-protective ventilation, a reduced pCO_2_ was observed in this study. Carbon dioxide is removed through both the healthy lungs and the extracorporeal membrane. The sweep gas flow on the membrane was also adjusted only every 30 min based on the blood gas analysis. Nevertheless, the pCO_2_ values were within the intended range.

Because the BE was within the normal range and no differences were observed between the groups, the elevated pH reflects the impaired pCO_2_. The priming of the ECMO circuit with unbalanced hydroxyl ethyl starch might explain the observed differences in electrolyte levels.

It is well known that the interaction of the endothelia cells with the large foreign surface of the ECMO membrane and the circuit results in ECMO-induced inflammation [5, 7]. Consequently, the elevated TNF-α levels might reflect ECMO-induced inflammation and are consistent with our previous studies [22, 23, 26]. However, no differences in TNF-α levels were observed during V-V or V-A ECMO therapy in septic rats compared to controls [10, 21]. Notably, TNF-α concentrations were threefold higher in septic rats compared to healthy rats [21]. Thus, the effect of the ECMO-induced inflammation appears minimal compared to that of lipopolysaccharide administration.

Patients during ECMO therapy are at high risk for nosocomial infection [6]. Frerou et al. investigated the immune alterations in humans with cardiogenic shock during V-A ECMO therapy [27]. After V-A ECMO initiation, a significant increase in circulating immature neutrophils and a lymphocyte dysfunction with an expansion of myeloid-derived suppressor cells were observed. In line with our study, elevated levels of TNF-α were measured. In contrast to our results, increased levels of IL-6 and IL-10 were seen [27]. These differences may reflect the fact that V-A ECMO therapy is applied in critically ill humans with pre-existing immune changes [7]. In contrast, our study was performed using healthy animals, limiting its applicability to human patients.

CXCL-2 can be produced by rat enterocytes and released after intestinal injury [28]. In addition, during lung inflammation, alveolar type II epithelial cells release CXCL-5 [29]. For these reasons, the authors decided to measure the concentrations of both chemokines. Again, the use of healthy rats may explain why the concentrations of CXCL-2 and CXCL-5 remained below 2 pg/mL (CXCL-2) and 20 pg/mL (CXCL-5), respectively.

Although all rats received lung-protective ventilation, the levels of the pro-inflammatory cytokine IL-6 were only reduced during high ECMO blood flow. Notably, blood flow through the native lungs depends on drainage from the right atrium, and faster blood flow through the ECMO system is associated with reduced blood flow through the native lungs, which may explain why IL-6 levels were only reduced during high ECMO blood flow. These observations are consistent with our previous study, in which increased pulmonary inflammation was observed only during low-flow ECMO therapy in a rat model of septic shock [10]. However, this increased pulmonary inflammation might be caused by a combination of high blood flow through the native lungs and lipopolysaccharide administration to induce septic shock. Again, since these findings in septic animals were not validated histological or by biomarkers, this conclusion remains speculative.

Nonetheless, this study had some limitations that should be acknowledged. Firstly, the findings of animal studies are not directly transferable to humans. Nonetheless, although rats have a faster heart rate than humans, they have a similar cardiopulmonary system. Moreover, V-A ECMO therapy is applied in critically ill patients with immune changes; therefore, our results in healthy rats cannot be transferred to critically ill patients. Secondly, the sample size is relatively small for multiple comparison, which might increase the risk of type I error. However, owing to animal welfare considerations, the local animal welfare commission required the authors to reduce the sample size to the minimum necessary to obtain meaningful results. Nevertheless, the study may have lacked sufficient power to detect smaller effect sizes. Thirdly, the baseline measurements were excluded from the statistical analysis. However, the primary aim of this study was to investigate the impact of V-A ECMO therapy on intestinal microcirculation; including baseline measurements without V-A ECMO support into the V-A ECMO groups would have skewed the results. Fourthly, the microcirculation of the mucosa should ideally be measured from the inside. However, micro-light guide spectrophotometry probes are designed to measure oxygenation at a depth of 2–4 mm, serving as a surrogate for intestinal microcirculation. Fifthly, local compression of the liver cannot be excluded due to the probe’s adhesive placement on the liver. Nonetheless, significant differences in the hepatic microcirculation were observed between sham and V-A ECMO therapy. Lastly, this study lacks histological validation and biomarker evaluation. Therefore, further studies should include histological validation and measurement of biomarkers like the intestinal fatty acid–binding protein.

## Conclusions

No alterations in intestinal microcirculation were observed between V-A ECMO therapy and sham animals in healthy rats. Despite ECMO-induced inflammation, reduced pulmonary inflammation was observed during high-flow V-A ECMO therapy and lung-protective ventilation in healthy rats.

## Methods

### Animals

All procedures involving animals were conducted in compliance with standards for animal care and the Animal Research: Reporting of In Vivo Experiments (ARRIVE) guidelines and approved by the Animal Welfare Commission of the Department of Veterinary Medicine at the Regional Council Giessen (GI 20/26 Nr. G 77/2019) [30].

Male Lewis rats (330–350 g) obtained from Janvier Labs (Le Genest-Saint-Isle, France) were housed at 22 °C, 55% relative humidity, and a 14/10-h day/night cycle, with ad libitum access to standard chow and water. The rats were randomly divided into three groups per lot to undergo V-A 60, V-A 90 or sham procedure (*n* = 10 per group). During sham procedure, all catheters were inserted, and the rats were monitored for 2 h without V-A ECMO therapy.

### Induction and maintenance of anaesthesia

Following inhalation induction of anaesthesia with 5% Isoflurane (Baxter, Unterschleißheim, Germany) balanced with 95% oxygen, the rats were intubated orotracheally (16 G cannula; B. Braun, Melsungen, Germany) and ventilated in a volume-controlled and weight-adjusted manner (tidal volume = 6.2 mL × body weight [kg]^1.01^, respiratory rate = 53.3 × body weight [kg]^−0.26^) with an inspiratory oxygen fraction of 0.5 using an Inspira ventilator (Harvard Apparatus, Cambridge, UK). Next, the rats were immobilised on a heating pad, controlled by rectal temperature measured with a rectal probe. Additional heat was applied with an infrared lamp to adjust the rectal temperature to 36.5 °C–37 °C. Then, the electrocardiograph was connected and the lateral tail vein was punctured percutaneously for continuous infusion of fentanyl (10 µg/kg/h; Albrecht GmbH, Aulendorf, Germany), midazolam (2 mg/kg/h; Roche, Basel, Switzerland), pancuronium (0.1 mg/kg/h; Inresa, Freiburg, Germany) and a balanced crystalloid solution (5 mL/kg/h, Sterofundin; B.Braun, Melsungen, Germany) [10, 21, 24, 26, 31].

### Cannulation and abdominal laparotomy

As previously described, the following vascular cannulas were placed after surgical preparation. First, the tail artery was punctured with a 24-G cannula (B. Braun, Melsungen, Germany) for continuous arterial blood pressure monitoring and intermittent blood gas analysis. After the groin was opened with a small skin incision, the femoral artery was dissected, cannulated with a 22-G cannula and connected to a three-way stopcock for return of ECMO blood. Next, the neck was opened with a small skin incision and the right internal jugular vein and carotid artery were dissected. Then, a 2F pressure–volume catheter (SPR-838; Millar, Houston, TX, USA) was carefully inserted through the carotid artery into the left ventricle to measure the SV, CO, LVEDV, LVEDP and LVEF.

After a median skin incision was made with surgical scissors, the abdominal cavity was opened via high-temperature electrocautery (AA01 Bovie high-temperature cautery; Bovie Medical Corporation, Clearwater, FL, USA). Next, the intestine was mobilised, and the vascular-free mesentery of a small intestine loop was dissected. Then, the probe for white light and laser Doppler spectrometry (LFX-151; LEA Medizintechnik GmbH, Heuchelheim, Germany) was placed on the intestine and secured with a one-side-open silicon tube (diameter: 8 mm) to ensure a loose fit. Next, the entire intestine was returned to its original position. Then, a shallow well was placed on the right lobe of the liver (LFX-45; LEA Medizintechnik GmbH, Heuchelheim, Germany), and the abdominal cavity was covered with a warm, wet compress [10, 21, 32].

Finally, all rats received heparin (400 IU/kg; Merckle GmbH, Blaubeuren, Germany), and the right internal jugular vein was cannulated with a custom modified multi-orifice 17-G cannula (B. Braun, Melsungen, Germany) for venous drainage to the ECMO circuit, as previously described in detail, and connected to a three-way stopcock [10, 24, 31].

### Extracorporeal membrane oxygenation

As previously described, the ECMO circuit consisted of a roller pump (Verderflex Vantage 3000; Castleford, UK), a venous reservoir (M. Humbs, Valley, Germany), and a membrane oxygenator (Micro-1; Kewei Rising Medical, Shenzhen, China) [10, 21, 24, 26, 31]. A Heidelberger extension line (B.Braun, Melsungen, Germany) was connected to a heating pump (HU35; Gettinge, Raststatt, Germany) and wrapped around the oxygenator to prevent heat loss. The draining cannula was connected to the venous reservoir by a shortened Heidelberg extension line (B.Braun, Melsungen, Germany), and the membrane oxygenator was connected to the return cannula by a shortened syringe pump line (B.Braun, Melsungen, Germany). A three-way stopcock (B.Braun, Melsungen, Germany) was connected to the venous reservoir for central venous blood gas analysis. The entire circuit was primed with 250 IU of heparin (Ratiopharm, Ulm, Germany) and 9 mL of 6% unbalanced hydroxyl ethyl starch (Voluven; Fresenius Kabi, Bad Homburg, Germany). Blood flow was initiated at 30 mL/kg/min and then continuously increased to the target flow of 60 (V-A 60) or 90 (V-A 90) mL/kg/min, respectively. The sweep gas flow through the membrane was adjusted between 15 and 60 mL/min to maintain a pCO_2_ of 35 – 45 mmHg. The oxygen fraction on the ECMO membrane was set to 0.5. After reaching target blood flow, lung-protective ventilation was achieved by adjusting the respiratory rate and tidal volume to 75% of the rat’s body weight using the following formulae: tidal volume = 6.2 mL × body weight (kg)^1.01^ and respiratory rate = 53.3 × body weight (kg)^−0.26^.

### Intestinal microcirculation

To access the intestinal and hepatic microcirculation, the intestinal and hepatic probes were connected to the micro-light guide spectrophotometry oxygen to see device (LEA Medizintechnik GmbH, Heuchelheim, Germany). As previously described, each probe consisted of two light sources and their corresponding optical sensors [10, 21, 32]. White light spectroscopy (450 – 1000 nm) was used to measure the percentage SO_2_, which is composed primarily of venous oxygen saturation and, secondarily, of arterial and capillary oxygen saturation. The amount of light absorption due to haemoglobin was analysed and expressed in relative haemoglobin arbitrary units (RUs). The second light source emitted laser light (820 nm, 30 mW) and was used to determine erythrocyte velocity and, thereby, relative blood flow.

### Time points of hemodynamic measurements

Baseline values were captured before initiation of V-A ECMO therapy. Subsequent measurements were taken every 10 min thereafter, up to 120 min.

### Blood analyses

Blood gas analyses were performed immediately before initiating V-A ECMO, then every 30 min up to 120 min (ABL800; Radiometer, Copenhagen, Denmark). Recordings consisted of S_a_O_2_, S_cv_O_2_, pO_2_, pCO_2_, haemoglobin, haematocrit, pH, BE, lactate, sodium, chloride, potassium, calcium, and glucose. Blood samples were collected for inflammation analysis immediately upon commencing V-A ECMO, then every 60 min up to 120 min. This blood was immediately centrifuged at 5000 rpm and 4 °C for 5 min, and the plasma samples were stored at − 80 °C for further analysis.

### End of experiments

After 120 min, isoflurane was adjusted to 5%, and the rats were euthanised by exsanguination via the returning and draining ECMO cannulas. Immediately after confirming death by asystole, the neck was opened, and the trachea was dissected. Next, a ligature was fixed around the trachea to seal the tube. Then, the lungs were flushed repeatedly with 40 mL of balanced crystalloid solution (Sterofundin; Fresenius, Bad Homburg, Germany). After centrifuging the BAL at 1200 rpm and 4 °C for 8 min, the supernatant was collected and stored at − 80 °C for further analysis [10, 21].

### Enzyme-linked immune sorbent assays

Systemic and pulmonary inflammation was assessed by the concentrations of the cytokines TNF-α, IL-6, IL-10, CXCL-2 and CXCL-5 in the plasma and BAL, measured using ELISAs (R6000B, RTA00 and R1000 from R&D System [Wiesbaden, Germany] and ERCXCL2 and ERCXCL5 from Thermo Fisher Scientific [Waltham, MA, USA]) according to the manufacturer’s instructions. Additional information about the ELISAs can be found in Table 2. The probes were only thawed once.

**Table 2:** Details about the ELISA

	**TNF-α**	**IL-6**	**IL-10**	**CXCL-2**	**CXCL-5**
Sensitivity [pg/mL]	5	36	10	2	20
Range [pg/mL]	12.5—800	62.5 – 4,000	31.2 – 2,000	2 – 1,800	20 – 6,000

CXCL-2 = C-X-C motif chemokine ligand 2; CXCL-5 = C-X-C motif chemokine ligand 5; IL-10 = interleukin 10; IL-6 = interleukin 6; TNF-α = tumour necrosis factor-alpha

### Statistical analyses

The required sample size was estimated using G*Power (version 3.1.9.2; Heinrich Heine University, Düsseldorf, Germany) with alpha and beta error rates of 0.05 and 0.02, respectively. Groups of 10 animals resulted in an effect size of 0.55 (moderate). An inbred rat strain was used to reduce variance between groups. Samples with measured concentrations below the detection limit of the ELISA were recorded as missing values and automatically excluded from further analyses.

All statistical analyses were performed using SPSS Statistics (version 20; IBM Corp., Stuttgart, Germany), and all graphs were created using GraphPad Prism (version 7; GraphPad Software, San Diego, CA, USA). A *p* < 0.05 was considered statistically significant. All data are presented as the median (interquartile range). Data were compared between groups using a repeated-measures analysis of variance followed by post-hoc Bonferroni tests. Because the baseline measurements were recorded without V-A ECMO support, they were excluded from this analysis.

## Supplementary Information

Supplementary Material 1

## Data Availability

The datasets used and/or analysed during the current study are available from the corresponding author on reasonable request.

## Abbreviations

ARRIVE: Animal research: reporting of in vivo experiments
BAL: Broncho alveolar lavage
BE: Base excess
Ca: Calcium
Cl: Chloride
CO: Cardiac output
CPB: Cardiopulmonary bypass
CXCL-2: C-X-C motif chemokine ligand 2
CXCL-5: C-X-C motif chemokine ligand 5
DAP: Diastolic arterial pressure
ECMO: Extracorporeal membrane oxygenation
ELISA: Enzyme-linked immunosorbent assay
Glu: Glucose
Hb: Haemoglobin
Hct: Haematocrit
IL-10: Interleukin 10
IL-6: Interleukin 6
K: Potassium
Lac: Lactate
LVEDP: Left ventricular end-diastolic pressure
LVEDV: Left ventricular end-diastolic volume
LVEF: Left ventricular ejection fraction
MAP: Mean arterial pressure
Na: Sodium
pCO_2_: Arterial partial pressure of carbon dioxide
pO_2_: Arterial partial pressure of oxygen
RU: Relative units
SaO_2_: Arterial oxygen saturation
SAP: Systolic arterial pressure
S_cv_O_2_: Central venous oxygen saturation
SO_2_: Tissue oxygen saturation
SV: Stroke volume
TNF-α: Tumour necrosis factor-alpha
V-A: Veno-arterial
V-V: Veno-venous
