# Supplementary material for: Hepatic and intestinal microcirculation and pulmonary inflammation in a model of veno-arterial extracorporeal membrane oxygenation in the rat
Source: Intensive Care Med Exp. 2026 Jul 7;14:88. doi: 10.1186/s40635-026-00938-w (PMC13341997; doi:10.1186/s40635-026-00938-w)
Supplement: Supplementary file 1 — Supplementary Material 1 [file 40635_2026_938_MOESM1_ESM.docx]

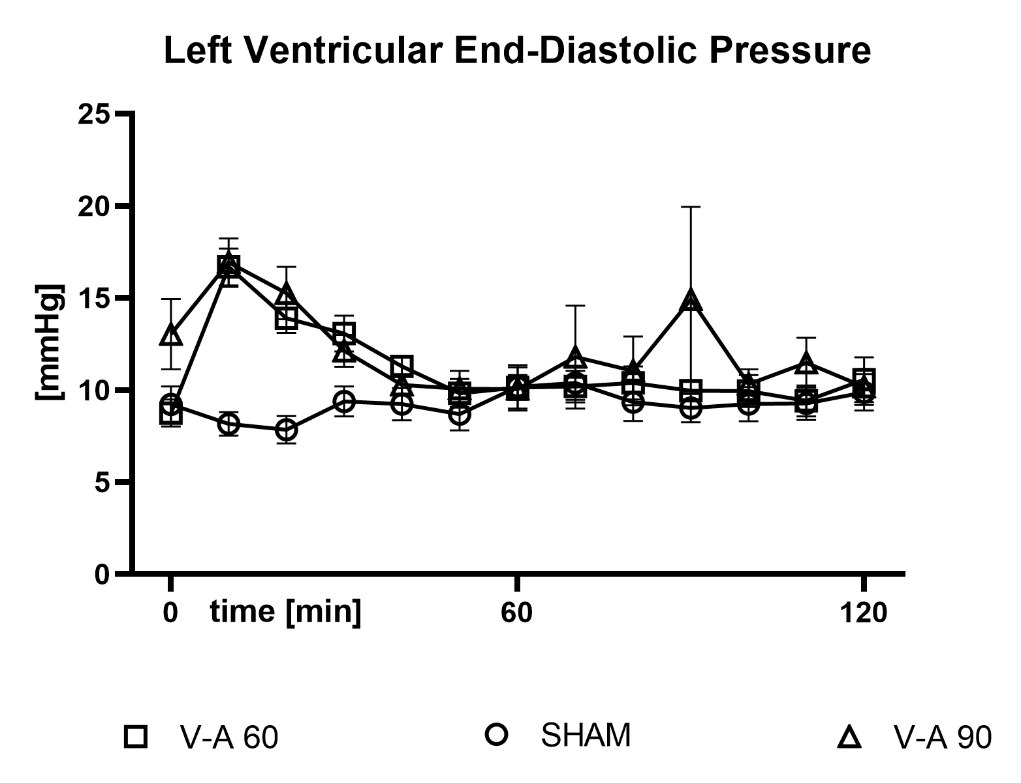


**Supplemental Figure 1:** Time course of LVEDP. LVEDP did not differ significantly between the groups (n = 10 per group). Abbreviations: V-A = veno-arterial.

**Supplemental Table 1:** Results of the Intestinal and hepatic microcirculation

|  |  | sham | V-A 60 | V-A 90 |
| --- | --- | --- | --- | --- |
| Intestinal | SO₂ (%) | 76 [72, 79] | 77 [71, 81] | 74 [72, 78] |
|  | Relative blood flow (RU) | 345 [302, 362] | 369 [343, 408] | 356 [339, 391] |
|  | Relative haemoglobin (RU) | 77 [72, 80] | 70 [69, 80] | 77 [72, 79] |
| Hepatic | SO₂ (%) | 43 [38, 48] | 23 [20, 31] | 27 [23, 38] |
|  | Relative blood flow (RU) | 101 [94, 103] | 91 [89, 92] | 93 [91, 97] |
|  | Relative haemoglobin (RU) | 193 [185, 213] | 208 [190, 221] | 224 [202, 265] |

Data are presented as the median [interquartile range]. Abbreviations: ECMO = extracorporeal membrane oxygenation; RU = relative units; SO_2_ = tissue oxygen saturation; V-A = veno-arterial.
